# The importance of adrenal hypoandrogenism in infertile women with low functional ovarian reserve: a case study of associated adrenal insufficiency

**DOI:** 10.1186/s12958-016-0158-9

**Published:** 2016-04-26

**Authors:** Norbert Gleicher, Vitaly A. Kushnir, Andrea Weghofer, David H. Barad

**Affiliations:** The Center for Human Reproduction, 21 East 69th Street, New York, NY 10021 USA; The Foundation for Reproductive Medicine, New York, NY USA; Stem Cell Biology and Molecular Embryology Laboratory, The Rockefeller University, New York, NY USA; Department of Obstetrics and Gynecology, Wake Forest University, Winston-Salem, NC 27106 USA; Vienna University School of Medicine, Vienna, Austria; Department of Obstetrics and Gynecology, Albert Einstein College of Medicine, Bronx, NY 10461 USA

## Abstract

**Background:**

Low testosterone (T), whether due to ovarian and/or adrenal insufficiency, usually results in poor follicle maturation at small growing follicle stages. The consequence is a phenotype of low functional ovarian reserve (LFOR), characterized by poor granulosa cell mass, low anti-Müllerian hormone and estradiol but rising follicle stimulating hormone. Such hypoandrogenism can be of ovarian and/or adrenal origin. Dehydroepiandrosterone sulfate (DHEAS) is exclusively produced by adrenals and, therefore, reflects adrenal androgen production in the zona reticularis. We here determined in a case study of infertile women with LFOR the presence of adrenal hypoandrogenism, its effects on ovarian function, and the possibility of presence of concomitant adrenal insufficiency (AI), thus reflecting insufficiency of all three adrenal cortical zonae.

**Methods:**

We searched our center’s anonymized electronic research database for women with LFOR, who were also characterized by peripheral adrenal hypoandrogenemia (total testosterone < 16.9 ng/dL) and low DHEAS (<76.0 μg/dL). Among 225 women with LFOR, we identified 29 (12.9 %). The adrenal function of so identified women were further investigated with morning cortisol and ACTH levels and/or standard ACTH stimulation tests. We also determined the prevalence of classical AI (insufficiency glucocorticoid production by zona fasciculata) in hypoandrogenic women with LFOR, and impact of adrenal hypoandrogenism on ovaries.

**Results:**

Among 14/28 women with adrenal hypoandrogenism due to insufficiency of the zona reticularis available for follow up, 4 (28.6 %) also demonstrated previously unrecognized classical primary, secondary or tertiary AI due to insufficiency of the zona fasciculata. An additional patient with presenting diagnosis of seemingly primary ovarian insufficiency (POI), demonstrated extremely low T and DHEAS levels, a diagnosis of Addison’s disease, and was on glucocorticoid but not androgen supplementation. As her dramatic improvement in ovarian function criteria after androgen supplementation confirmed, her correct diagnosis, therefore, was actually secondary ovarian insufficiency (SOI) due to adrenal hypoandrogenism.

**Conclusions:**

Women with LFOR, characterized by low T and DHEAS, are also at risk for AI, while women with AI may be at risk for adrenal induced hypoandrogenism and, therefore, SOI. A currently undetermined percentage of POI patients actually are, likely, affected by SOI, a for prognostic reasons highly significant difference in diagnosis.

## Background

For many years hypoandrogenemia has been recognized as a characteristic feature of primary ovarian insufficiency (POI) [1]. More recently, low testosterone (T) levels have also been reported in association with milder cases of POI, so called occult POI (oPOI), characterized by low (age specific) functional ovarian reserve (LFOR) [2].

Over the last decade various animal models and clinical human experience have provided increasing evidence that T is essential for normal follicle growth and maturation during small growing follicle stages. Insufficient androgen receptor (AR) activity on granulosa cells leads to poorer growth of fewer follicles, and to poor oocyte quality in surviving follicles [3].

Albeit still controversial [4, 5], these observations have led to androgen supplementation in women with hypoandrogenic LFOR [6], and to the suggestion that pregnancy success with in vitro fertilization (IVF) in hypoandrogenic LFOR directly correlates with improvements in patients’ testosterone levels [7].

Ovaries (theca cells) and adrenals (zona reticularis) produce the majority of androgens. Consequently, like hyperandrogenism in association with polycystic ovary syndrome (PCOS) [8], hypoandrogenism can be of ovarian and/or adrenal etiology. Though accurate differentiation is not always possible, it is generally accepted that low dehydroepiandrosterone sulfate (DHEAS), almost exclusively produced by the zona reticularis of adrenals, in association with low testosterone levels, strongly suggests adrenal origin of low androgen levels [9–11].

We recently discovered that, as reflection of adrenal function, peripheral androgen precursor levels in infertile women with LFOR correlate with morning cortisol [12]. This observation suggests that adrenal and ovarian functions may to a degree be interdependent. Such interdependency is also supported by the common embryonic primordium of adrenals and ovaries [13].

We, therefore, in this study investigated this interdependence of adrenals and ovaries based on the recent recognition that LFOR, independent of cause, is usually characterized by peripheral hypoandrogenemia [2]. Based on the presumed origin of patients’ hypoandrogenemia, we then further assessed adrenal function under the hypothesis that adrenal origin of hypoandrogenemia (zona reticularis) may also raise the specter of adrenal insufficiency (AI) in the other two layers of the adrenal cortex.

As further evidence for the hormonal interrelationship of adrenals and ovaries, we here report four cases of previously unknown AI in hypoandrogenic women with LFOR and one case of known primary AI (Addison’s disease), which was treated with glucocorticoid but not androgen supplementation and, therefore, presented with secondary ovarian insufficiency (SOI) due to AI This case had previously been erroneously diagnosed as primary ovarian insufficiency (POI).

## Methods

Our center maintains an anonymized electronic research database, which includes patients who consent to use of their medical records for research purposes as long as those remain confidential and the patients’ anonymity is maintained. Use of this electronic database was approved by our center’s IRB for this study (IRB of The Center for Human Reproduction, Neil Rosenberg, MD, Chairman, IRB application number ER0330215/01). All patients reported here gave written consent for use of their medical records.

We identified in this database 225 infertile patients with LFOR, defined as follicle stimulating hormone (FSH) above, and/or anti-Müllerian hormone (AMH) below, age-specific 95 % CI [14, 15]. To identify women with potential adrenal hypoandrogenism, we further searched among LFOR patients for those with abnormally low total testosterone (TT). We selected TT rather than free testosterone (FT) to define the study population because TT levels have been demonstrated to marginally better reflect IVF outcomes than FT [7].

Abnormally low TT was defined as the lower third of normal laboratory range (<16.0 ng/dL)], while abnormally low DHEAS was defined as below the 15^th^ percentile of normal laboratory range (<76 μg/dL). Both cut offs have been used in the context of LFOR investigations before [2, 12]. All androgen assays were performed utilizing liquid chromatography/tandem mass spectrometry.

Among 29 women with low TT and DHEAS, 14 were available for follow up assessments of adrenal function with morning cortisol (C) and adrenocorticotropin (ACT) levels and/or full 2-h ACTH stimulation tests. The other 15 patients either could not be reached or refused participation in this follow up.

Morning C was considered abnormally low at levels of < 5.0 μg/dL, while ACTH was considered abnormally high at > 100 pg/mL. C and ACTH levels were obtained by commercial assays. Adrenocorticotropin hormon stimulation was performed with Cortrosyn® (cosyntropin, Amphastar Pharmaceuticals, Inc), 0.25 mg in routine fashion, with cortisol levels determined at 30 and 60 min. and recently described [16, 17].

Adrenal insufficiency is defined as the inability of the adrenal cortex to produce sufficient amounts of glucocorticoids and/or mineralocorticoids [16, 18]. Abnormally low cortisol, by lowering feedback, induces increased stimulation of the adrenal cortex by (ACTH), which disrupts adrenal production of mineralocorticoids, leads to increased plasma renin release by the juxtaglomerular cells of the kidney and the well-known symptomatology of primary AI. Abnormally high ACTH concentrations are important not only because of their disruptive effects on the adrenal cortex but also because they allow differentiation of primary from secondary AI, with the latter characterized by abnormally low ACTH levels and, therefore, absence of secondary clinical effects of excessive ACTH stimulation [18].

## Results

Out of 14 patients identified with likely adrenal hypoandrogenism, 10 were found to demonstrate entirely normal adrenal function. Table 1 summarizes the clinical presentations of four who as part of this study for the first time received a diagnosis of AI. One patient was diagnosed with primary AI, two were diagnosed with secondary AI and a fourth with tertiary AI.

**Table 1:** Characteristics of 4 patients diagnosed with previously unknown AI among women with adrenal hypoandrogenemia*

Patient	Age (years)	Diagnoses				Laboratory				Final diagnosis
		Primary	Other	Immune	FSH (mIU/ML)	AMH (ng/mL)	Androgens	ACTH (pg/mL)	Cortisol (ug/dL)	
1	28	POI^1^	Hypothyroid	+TPO^2^	30.0	3.04	TT 7.0 ng/dL	464.7	8.8	Primary AI
				+ TG^3^	14.4	2.12	TT ud^6^			
			Celiac	+ DG^4^			DHEA ud			
				+ TG^5^			DHEAS ud			
2	30	PCOS	SLE^7^		8.0	2.68	FT 0.4 pg/mL	**		Likely iatrogenic AI
			HNA^8^				TT 17.0 ng/dL			
							DHEA 273.0 ng/dL			
							DHEAS ud			
	***					4.11	TT 30.0 ng/dL			
							DHEA 272.0ug/dL			
3	30				7.7	ud	DHEAS 70.0ug/dL	ud	1.4	Secondary AI as part of pan- hypo-pituitarism
					8.7	ud	FT 1.4 pg.mL		1.7	
							TT 14.0 ng/dL			
							DHEAS 56ug/dL			
4	46		Crohn’s		12.5	ud	TT ud	ud	1.4	Secondary AI
			Hypothyroid				FT ud			
							DHEA 117.0ug/dL			
							DHEAS 13.0ug/dL			

^1^ *POI* primary ovarian insufficiency; ^2^ *TPO* thyroid peroxidase antibody; ^3^ *TG* thyroglobulin antibody; ^4^ *DG* deamidated gliadin antibody (IgA); ^5^ *TG* t-transglutaminase (igA) antibody; ^6^ *ud* undetectable; ^7^ *SLE* systemic lupus erythematosus treated with 7 mg prednisone p.o. o.d.; ^8^ *HNA* non-heredetary angioedema

*Only 14 of 29 women identified in the center’s research database with adrenal hypoandrogenemia have so far been investigated in follow up

** Not obtained since patients received long-term prednisone

*** Androgens and AMH level after supplementation with DHEA

*Patient 1:* Upon diagnosis with PAI, the patient initiated supplementation with hydrocortisone, and continued her supplementation with levothyroxine. Though this patient presented to our center with a diagnosis of POI, her FSH values did not support this diagnosis but a diagnosis of oPOI/POA

*Patient 2:* This patient consulted long-distance with our center after a spontaneous pregnancy loss in a spontaneously conceived pregnancy and after an IVF cycle suggestive of PCOS (29 oocytes), but with only 2 poor-quality embryos. After low androgens were noted, we recommended supplementation with DHEA 25 mg p.o., t.i.d. Androgen levels improved, as did her AMH, and the patient spontaneously conceived what was diagnosed as an ectopic pregnancy. We suspect this to represent a case of iatrogenic (tertiary) AI, secondary to prolonged prednisone supplementation

*Patient 3:* This patient presented to our center since us of a gestational carrier had been recommended to her elsewhere

*Patient 4:* This patient presented with primary infertility and Crohn’s disease, treated with Enbrel® (etanercept)

The case of tertiary AI was a long standing systemic lupus erythematosus (SLE) patient on corticosteroid therapy who also suffered from long standing infertility. Though she had failed a prior in vitro fertilization (IVF) cycle, she spontaneously conceived once her androgens were supplemented with dehydroepiandrosterone (DHEA) normalizing her abnormally low TT and excessively high sex hormone-binding globulin (SHBG) levels.

We in addition identified a 41-year-old female with established Addison’s disease (primary AI) on glucocorticoid replacement who was not being supplemented with androgens. She based on a greatly elevated FSH level of 44.0 mIU/mL presented with diagnosis of POI. Her TT, FT, DHEA and DHEAS were all below lower cut off levels of normal range, while her SHBG was above normal range. She, thus, with great likelihood demonstrated adrenal hypoandrogenism. Once supplemented with DHEA (25 mg TID), her androgens normalized and her FSH level declined to 14.8 mIU/mL.

This patient’s presenting diagnosis of POI was, therefore, incorrect; a consequence adrenal hypoandrogenism of the zona reticularis, she really suffered from SOI, which apparently was part of a mixed adrenal cortex insufficiency of the zona fasciculata (leading to Addison’s disease) and the zona reticularis (leading to SOI).

## Discussion

Adrenal insufficiency (AI) is a complex, at times life threatening disease, which can be the result of failure of the adrenal glands (primary AI), can be consequence of failure of the hypothalamic/pituitary axis (secondary AI) or can be iatrogenic (tertiary AI) [16, 18]. We here report on four (out of 14) women with adrenal hypoandrogenism (low T and low DHEAS), who, upon adrenal evaluation, were found to suffer from AI, -one case of primary, two cases of secondary and one case of tertiary AI.

We also discovered among those patient one woman who already had been diagnosed with Addison’s disease (primary AI) and received glucocorticoid supplementation. Her treating physician was, however, unaware that, due zona reticularis insufficiency, she also was severely hypoandrogenic. With definition of AI currently restricted to insufficiency of zona fascilulata (glucocorticoids) and zona glomerulosa (mineralocorticoids), adrenal hypoandrogenism of the zona reticularis is, interestingly, excluded from the diagnosis of AI [16, 18].

**Fig. 1:**
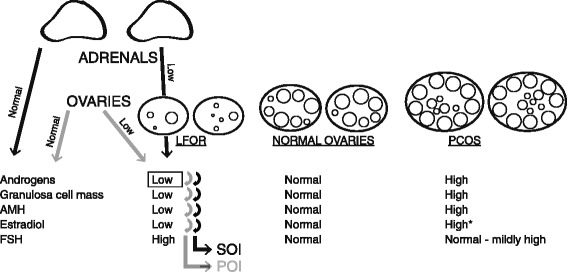
Control of ovarian function via adrenal androgen production. *Under ovarian stimulation with gonadotropin very high; The figure depicts how androgen production in the zona reticularis of the adrenals can affect ovarian function: With low androgen production (for example insufficient capacity to sulfonize DHEA to DHEAS), growth and development of small growing follicles in ovaries (also called the functional ovarian reserve, FOR) is inhibited. LFOR develops, resulting in low estradiol and AMH levels due to declining granulosa cell mass. Due to decreasing feedback via estradiol, FSH production in the pituitary increases, leading to typically elevated FSH levels, usually attributed to a POI phenotype. These cases, however, actually represent a secondary form of ovarian insufficiency (SOI), primarily driven by adrenal hypoandrogenism. At the opposite extreme of ovarian function, androgen production by the adrenal cortex is responsible for some cases of PCOS

In presence of severe hypoandrogenism (all of her androgen values were significantly below the lower cut offs of normal range), testosterone-dependent small growing follicle stages [3], likely, arrested. As a consequence, granulosa cell mass and estradiol production declined, AMH levels dropped and FSH increased due to diminished feed back on the pituitary, leading to what can be easily mistaken for a fairly typical clinical POI phenotype (Fig. 1). Due to its adrenal origin, it, however, really represents a form of SOI.

With 4/14 women diagnosed with a form of AI, we here report an unexpectedly high, and likely exaggerated prevalence of AI in infertile women with hypoandrogenic LFOR of adrenal etiology. Further, larger scale studies are obviously needed to assess the true prevalence. However, even here presented small case series suggests that in such women a careful adrenal evaluation appears indicated.

Here presented data in addition suggest that AI may not only include insufficiencies of zona fasciculate and zona glomerulosa but also also of the zona reticularis, leading to adrenal hypoandrogenism. Therefore, one also has to conclude that, at least in women of reproductive years, assessments of peripheral androgens appear indicated if AI is suspected or already diagnosed.

POI is classically associated with hypoandrogenism [1, 2], usually primarily the consequence of insufficient T production of ovarian theca cells. POI, therefore, represents true ovarian insufficiency/aging, while in cases of SOI, once adrenal hypoandrogenism is appropriately supplemented, ovarian function may normalize, and responses to pharmacological stimulation of ovaries may improve. Figure 1 explains the underlying pathophysiology.

Correct differential diagnoses between POI and SOI are, therefore, of importance since a diagnosis SOI often reflects a much better prognosis. We, in SOI, indeed, have seen relative normalization of abnormally high FSH levels (Patient 2 in Table 1 and above noted patient with previously known Addison’s disease), and in Patient 2 even encountered a spontaneous pregnancy following normalization of T and SHBG levels after DHEA supplementation. Such radical changes for the better in ovarian phenotype will practically never be witnessed in cases of POI.

As in women with polycystic ovarian syndrome (PCOS), relative contributions to hyperandrogenisms of adrenals and ovaries are at times difficult to separate [8], so are in women with LFOR contributions to low T. During steroidogenesis, DHEA is via sulfotransferase converted to DHEAS (its 3β-sulfate), a conversion almost exclusively coded by the *SULT2A1* gene. Sult2A1 is, however, highly expressed only in the zona reticularis of the adrenal cortex, and practically absent in ovarian tissue [9–11]. If DHEAS is also abnormally low, low T in women can, therefore, be assumed to be of adrenal origin.

A few more words about the relevance of AI to ovarian function: Though the underlying pathophysiology for this association has remained unresolved, primary AI has been associated with female infertility for decades. [19]. Here suggested pathophysiology of SOI offers a possible explanation.

In developed countries over 90 % of primary AI is considered autoimmune in etiology, frequently coexisting with other autoimmune endocrinopathies [18, 20]. Interestingly, the only so far histopathologically defined autoimmune condition of ovaries in humans, so-called autoimmune oophoritis, practically exclusively only occurs in association with primary AI. This dependency strongly suggests common immunologic epitopes in adrenals and ovaries as targets of shared autoimmune attacks, possibly representing steroidogenic enzymes [21, 22]. Autoantibodies to 21-hydroxylase are widely considered diagnostic of primary AI, and are accepted as evidence for the autoimmune etiology of AI. These autoantibodies, indeed, often precede diagnosis of the disease [18, 23], and approximately 30 % of asymptomatic individuals with positive 21-hydroxylase antibodies will progress to clinical primary AI within five years [24].

Why is this important within here presented discussion?

Addison’s disease can also occur in absence of 21-hydroxylase antibodies, though such cases are rare except in young children and the elderly [15]. Other than 21-hydroxylase, yet unknown antibodies to steroidogenic enzymes and/or other common epitopes between adrenals and ovaries may, therefore, also play an important role in anti-adrenal and anti-ovarian autoimmunity. Autoantibodies to other steroidogenic enzymes have, indeed, been reported [22], though, currently are not considered diagnostic of primary AI.

Autoimmunity to endocrine glands is a commonly observed phenomenon; many such attacks, indeed, occur in combinations [20]. It, therefore, is difficult to imagine that anti-adrenal autoimmunity can affect only zona fasciculate and zona granulosa but will not affect the zona reticularis. For that reason, it appears somewhat puzzling that an autoimmune attack on the androgen-producing zona reticularis is currently not considered a possibility in AI. It appears more reasonable to assume that all three zones of the adrenal cortex can be subjects of autoimmune attacks, sharing risks and manifesting different combinations of involvement. Patients with glucocorticoid deficiency for that reason have to be monitored for the development of mineralocorticoid deficiencies [18]. Here presented study supports the contention that the zona reticularis should also be considered as a potential target of adrenal autoimmunity in presence of autoimmune attacks against the other two zonae.

In the developed world, in absence of tuberculosis and trauma, AI is, practically universally, considered an autoimmune condition [25]. Low T due to adrenal causes, therefore, with great likelihood has also to be considered autoimmune.

Finally, here discussed findings may also have relevance for the PCOS, increasingly viewed as the opposing extreme of LFOR on a spectrum of ovarian function [20]: While LFOR is hypoandrogenic [1, 2] and associated with low follicle yields, PCOS typically presents with high T, excessive follicle recruitment and, therefore, high FOR. At excessively high T levels, follicles, however, arrest at preantral stages [26]. Adrenal androgen production thus influences ovarian function over a wide range, from SOI to secondary PCOS (Fig. 1).

## Conclusions

We, therefore, conclude that, functionally, adrenals and ovaries have increasingly to be viewed as a single endocrine unit. The common developmental primordium (primordial germ cells) of adrenals and gonads is supportive of such a concept [13].

This study further strengthens recently reported evidence that adrenal glands and ovaries closely interact in regulating ovarian function via shared androgen production, which, in turn, affects follicle maturation in ovaries. This study, in addition, however, for the first time also demonstrates mutual diagnostic dependencies: LFOR in women of reproductive age, especially if associated with relative hypoandrogenism of adrenal origin (characterized by low DHEAS), should be seen as an indication for adrenal function evaluation. Concomitantly, a diagnosis of AI in women in reproductive years should immediately be considered an indication for evaluation FOR in the patient.

## Abbreviations

ACTH: adrenocorticotrophic hormone
AI: adrenal insufficiency
AMH: anti-Müllerian hormone
C: cortisol (morning)
DHEA: dehydroepiandrosterone
DHEAS: dehydroepiandrosterone-sulfate
FOR: functional ovarian reserve
FT: free testosterone
IVF: in vitro fertilization
LFOR: low functional ovarian reserve
PAI: primary adrenal insufficiency
PCOS: polycystic ovary syndrome
POI: primary ovarian insufficiency
POA: premature ovarian aging
SHBG: sex hormone-binding globulin
SOI: secondary ovarian insufficiency
T: testosterone
TT: total testosterone
